# Evaluation of antibiotic susceptibility in wound infections: A pilot study from Bangladesh

**DOI:** 10.12688/f1000research.12887.1

**Published:** 2017-12-07

**Authors:** Sushmita Roy, Mejbah Uddin Ahmed, Bhuiyan Mohammad Mahtab Uddin, Zubair Ahmed Ratan, Monali Rajawat, Varshil Mehta, Sojib Bin Zaman

**Affiliations:** 1 Department of Microbiology, Enam Medical College, Savar, Dhaka, Bangladesh; 2 Department of Biomedical Engineering, Khulna University of Engineering and Technology, Khulna, Bangladesh; 3 Department of Internal Medicine, RNT Medical College, Udaipur, India; 4 Department of Internal Medicine, MGM Medical College, Navi Mumbai, India; 5 Institute of Tropical Medicine and International Health, Berlin, Germany

**Keywords:** wound infection, bacterial pathogen, antibiotic susceptibility pattern

## Abstract

**Introduction**: Infections due to antibiotic resistant bacteria have increased alarmingly in both developed and developing countries. Unrestrained and rapidly spreading bacterial growth has turned the management of wound infections into a serious challenge. This study aimed to determine the prevalence of different bacterial pathogens and their antibiotic susceptibility in various types of wound infections.

**Methods**:  A cross-sectional study was conducted to collect 105 wound swabs. All isolated bacteria were identified based on colony characteristics, gram stain and standard biochemical tests, and antibiotic susceptibility testing (AST) with the disc diffusion method. Descriptive statistics were used to present the study findings, and all analyses were performed using Stata Version 13.

**Results**:  The rate of isolation of bacteria was 92.3%. *Staphylococcus aureus* was found to be the most frequent isolate (55.7%), followed by *Escherichia coli* (23.7%), *Pseudomonas* spp. (8.2%), and *Streptococcus pyogenes* (7.2%). Gram-positive bacteria were mostly (60%) found sensitive to vancomycin, azithromycin, gentamicin, imipenem, cefixime, and ceftriaxone in this study. Among the Gram-negative bacteria, *Escherichia coli* (>60%) showed sensitivity to cefixime, azithromycin, cefuroxime, ceftriaxone, cefotaxime, gentamycin, and ceftazidime.

**Conclusions**: The diversity of isolated bacteria and their susceptibility patterns signify a need to implement a proper infection control strategy, which can be achieved by carrying out antibiotic sensitivity tests of the isolates.

## Introduction

Wounds follow the loss of skin integrity, which provides a moist, warm and nutritive environment that is known to be conducive to microbial colonization and proliferation ^1^. Wound infections are considered a major complication of surgery, and can be classified into three types: incisional surgical wounds, deep incisional wounds, and organ-specific infections ^2^. Despite maintaining the high standards of preoperative preparation, antibiotic prophylaxis, and operative procedures, the appearance of postoperative wound infections remains a grave threat among the clinicians ^3^. Some of the most frequent causative microorganisms are related to wound infections and include *Staphylococcus aureus*, *Streptococcus pyogenes*, *Enterococci*, *Escherichia coli, Klebsiella pneumonia*, *Proteus* species and *Pseudomonas aeruginosa*. However, the severity of complication is largely based on the virulence of the infecting pathogen and the site of infection ^4^. The reporting trend of infection varies depending on the surgeon’s ability, operative area, surgical procedures, patient characteristics, etc. For instance, approximately 5,00,000 infections per year take place in the United States among an estimated 27 million surgical procedures ^5^. The incidence of hospital-based postoperative infection varies from 10%–25% in India ^6^. Nosocomial infection is becoming a serious problem affecting hospitalized patients both in developed and developing countries. According to a study conducted in Bangladesh, it was reported that among 38% of nosocomial infections, more than 50% were due to wound infection ^7^. Moreover, wound infections were found to be higher (49%) among post-operative patients as compared to pre-operative patients (15.9%) in that study ^7^. Post-operative wound infections have emerged as one of the important causes of morbidity among the hospitalized patients ^8^. Emmerson *et al*. reported that surgical wound infections account for 12.3% of all hospital-acquired infections ^9^. Wound infection is becoming a major concern among patients and healthcare practitioners for its increased toll on morbidity and financial loss. It also generates demand for attaining expensive management within the public health system ^5^. Active and passive surveillance of surgical site infections in the hospital will help the surgeons and clinicians to know the antibiotic susceptibility pattern related to the surgical site, which can help reduce postoperative complications ^10^.

The present study aimed to collect data on the bacteriological profiles of wound infections and their antibiotic susceptibility patterns in a teaching hospital in Bangladesh.

## Methods

### Study design and study timeline

This cross-sectional study was conducted from the 10 ^th^ of July 2016 to the 30 ^th^ December 2016.

### Study participants

105 samples of pus or wound swab were collected from the Microbiology Department of the Enam Medical College Hospital, Dhaka, which is a teaching hospital located in Bangladesh. The Microbiology department collected the samples from the outpatient and inpatient department of Surgery, Medicine, Gynaecology, and Orthopaedic.

### Data collection

105 swab samples were collected from patients with various wound infections including post-operative surgical wounds, burn wounds and superficial and soft tissue infections (SSTI) by paramedics. Selective criteria were considered: infected wound, adult patients, and before administration of antibiotics. Specimens were collected aseptically by nurses or technicians before the wound cleaning and before application of an antiseptic solution. At the time of swab collection, standard care was taken to avoid contamination by the normal flora of the surrounding skin. Then the specimens were transported within one hour to the Microbiology laboratory of the hospital to perform the culture and susceptibility tests. Subsequently, each specimen was inoculated on appropriate agar media: blood agar, MacConkey’s agar, nutrient agar, and mannitol salt agar media. Finally, the cultures were incubated aerobically at 37°C for 24–48 hours with proper care. All the plates were regularly inspected for growth, and identification of the isolated bacteria was done by colony morphology, gram-staining and standard biochemical tests by microbiologists ^11^. Antimicrobial susceptibility patterns of the isolated bacterial pathogens were tested by using commonly used antibiotics such as amoxicillin (10 µg), penicillin (10 µg), vancomycin (30 µg), azithromycin (15 µg), cephradine (30 µg), tetracycline (30 µg), cloxacillin (5 µg), co-trimoxazole (23.75 µg), ciprofloxacin (5 µg), cefixime (5 µg), cefuroxime (30 µg), imipenem (10 µg), ceftriaxone (30 µg), and nitrofurantoin (300 µg) using the Kirby Bauer disc diffusion method according to the guidelines of Clinical Laboratory Standards Institute ^12^.

### Statistical analysis

Errors in data were revised after cross-checking the laboratory records and clinical case recording forms. Descriptive statistics were used to interpret the data. Frequency and proportions were used to present categorical variables while mean and standard deviation (SD) were given to describe continuous variables. Stata (v.13) was utilized to analyze the data.

### Ethical statement

Written informed consent was obtained from each participant. All study participants were informed verbally about the objective of the study. The research team paid the costs related to patient sample collection. The study was conducted under the clearance of the Ethical Review Committee (approval# 2017/218) of Enam Medical College Hospital, Dhaka, Bangladesh.

## Results

### Characteristics of study participants

**Figure 1.**
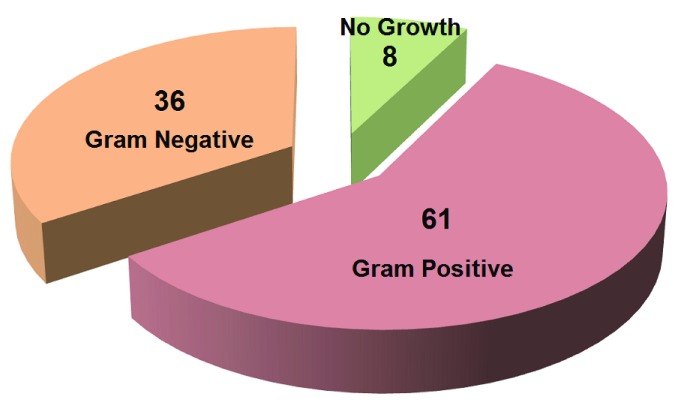
Pattern of bacterial growth among total samples (N=105). In this figure, red, magenta, and green portion indicates the Gram Positive, Gram Negative, No growth, respectively and indicates the percentage of bacterial growth.

The mean (±standard deviation) age of the study participants was 37 (±08) years, and 57.1% of participants were male. The rate of isolation of bacteria was 92.3%. Figure 1 shows the frequency of bacterial growth. Around 62.9% of culture positive plates turned out to be Gram-positive organisms, and 37.1% Gram-negative (n=97). Only 7.6% did not yield any growth in a culture plate.

### Isolation of different types of bacteria

**Figure 2.**
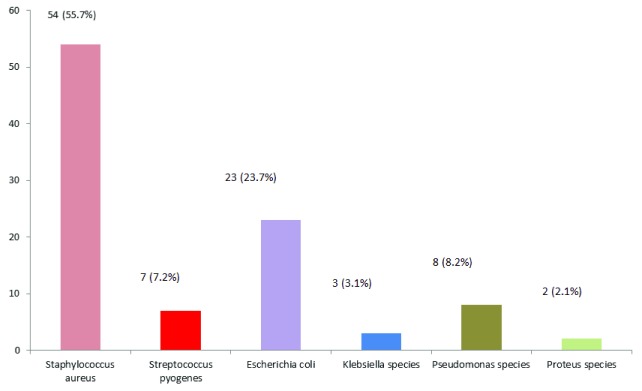
Rate of isolation of different bacteria (N=97). Rate of isolation of different bacteria are mentioned here based on number and their corresponding percentage.

*Staphylococcus aureus* (n=54; 55.7%) was predominantly found to be isolated among all the presenting bacteria. The frequency of *Escherichia coli* and *Pseudomonas* species was 23.7% and 8.2%, respectively ( Figure 2).

### Sensitivity pattern of isolated Gram-positive and Gram-negative bacteria

The susceptibility pattern of Gram-positive bacteria was mostly isolated to imipenem (90%), followed by ceftriaxone (85.5%), gentamycin (81.8%), vancomycin (80.8%), azithromycin (76.5%) and other antibiotics (<75.0%) ( Table 1).

**Table 1.** Sensitivity pattern of isolated Gram-positive bacteria (N = 61).

Antimicrobial agents	*Staphylococcus* *aureus* (n=54)	Streptococcus pyogenes (n=7)
Amoxicillin (10 µg)	32 (59.3%)	4 (57.1%)
Penicillin (10 µg)	30 (55.6%)	4 (57.1%)
Vancomycin (30 µg)	41 (75.9%)	6 (85.7%)
Azithromycin (15 µg)	44 (81.5%)	5 (71.5%)
Cephradine (30 µg)	32 (59.3%)	4 (57.1%)
Tetracycline (30 µg)	32 (59.3%)	4 (57.1%)
Cloxacillin 5( µg)	31 (57.4%)	4 (57.1%)
Co-trimoxazole (23.7 µg)	31 (57.4%)	3 (42.9%)
Gentamicin (10 µg)	42 (77.8%)	6 (85.7%)
Ciprofloxacin (5 µg)	32 (59.3%)	4 (57.1%)
Cefixime (5 µg)	40 (74.1%)	5 (71.5%)
Cefuroxime (30 µg)	32 (59.3%)	4 (57.1%)
Imipenem (10 µg)	51 (94.4%)	6 (85.7%)
Ceftriaxone (30 µg)	46 (85.2%)	6 (85.7%)

Most of the Gram-negative isolates were sensitive to ceftazidime (79.0%), ceftriaxone (71.8%), gentamicin (70.7%) and other antibiotics (<70.0%) ( Table 2). Most of the *Pseudomonas* spp. (>50%) were sensitive to ceftriaxone, imipenem, and gentamycin.

**Table 2.** Sensitivity pattern of isolated Gram-negative bacteria (N = 36).

Antimicrobial Agents	Escherichia coli (n=23)	*Klebsiella* spp. (n=3)	*Pseudomonas* spp. (n=8)	Proteus spp. (n=2)
Cephradine (30 µg)	10 (43.5%)	0 (0.0%)	0 (0.0%)	0 (0.0%)
Co-trimoxazole (23.7 µg)	12 (52.2%)	1 (33.3%)	1 (12.5%)	1 (50.0%)
Cefixime (5 µg)	19 (82.6%)	1 (33.3%)	1 (12.5%)	1 (50.0%)
Penicillin (10 µg)	8 (34.8%)	0 (0.0%)	0 (0.0%)	0 (0.0%)
Aztreonam (30 µg)	17 (73.9%)	1 (33.3%)	1 (12.5%)	2 (50.0%)
Cloxacillin (5 µg)	11 (47.8%)	0 (0.0%)	0 (0.0%)	0 (0.0%)
Cefuroxime (30 µg)	18 (78.3%)	0 (0.0%)	0 (0.0%)	2 (100%)
Tetracycline (30 µg)	14 (60.9%)	0 (0.0%)	3 (37.5%)	1 (50.0%)
Imipenem (10 µg)	21 (91.3%)	1 (33.3%)	4 (50.0%)	2 (100%)
Ceftriaxone (30 µg)	21 (91.3%)	1 (33.3%)	5 (62.5%)	2 (100%)
Ciprofloxacin (5 µg)	5 (21.7%)	0 (0.0%)	3 (37.5%)	1 (50%)
Azithromycin (15 µg)	8 (34.8%)	2 (66.7%)	3 (37.5%)	2 (100%)
Amoxicillin (10 µg)	1 (4.3%)	1 (33.3%)	0 (0.0%)	0 (0.0%)
Cefotaxime (30 µg)	20 (86.9%)	1 (33.3%)	0 (0.0%)	1 (50%)
Gentamycin (10 µg)	19 (82.6%)	3 (100%)	4 (50%)	1 (50.0%)
Ceftazidime (30 µg)	18 (78.3%)	3 (100%)	3 (37.5%)	2 (100%)
Nitrofurantoin (300 µg)	18 (78.3%)	1 (33.3%)	1 (12.5%)	1 (50.0%)

Patient characteristics Age, sex, residence, occupation, blood pressure, and diabetic mellitus status are given. Click here for additional data file. Copyright: © 2017 Roy S et al. 2017 Data associated with the article are available under the terms of the Creative Commons Zero "No rights reserved" data waiver (CC0 1.0 Public domain dedication).

Antibiotic susceptibility of bacterial cultures Includes data on *S. aureus, S. pyogenes, E. coli, Klebsiella spp, Pseudomonas,* and *Proteus.*
Copyright: © 2017 Roy S et al. 2017 Data associated with the article are available under the terms of the Creative Commons Zero "No rights reserved" data waiver (CC0 1.0 Public domain dedication).

## Discussion

Management of post-operative wound infection remains a significant concern for physicians globally ^13^. The problem has magnified due to the rapidly spreading resistance to the available array of antimicrobial agents ^14, 15^. We found that Gram-positive organisms accounted for 62.9% of isolates, compared to Gram-negative isolates that accounted for 37.1%. *Staphylococcus aureus* (55.7%) was the major microbial pathogen responsible for the wound infections. According to Centre for Disease Control and Prevention (CDC), *Staphylococcus aureus* is the most common organism associated with surgical wound infections. This study supports the results reported by Nwachukwu *et al*. ^16^, where 42.3% of infections were found to be caused by *Staphylococcus aureus*. Among the Gram-negative organisms, *Escherichia coli* were frequently isolated (23.7%) in our study. This finding is in line with a previous study which identified *Escherichia coli* as the major pathogen in the wound infection, followed by *Staphylococcus aureus* in a different setup ^17^. A previous survey conducted in Lahore supported our findings demonstrating that *Staphylococcus aureus* was the main causative organism of surgical infection ^18^.

In our study, we found imipenem as the most active antibiotic, with a susceptibility of 94.4% against *Staphylococcus aureus*. This study showed high sensitivity of *Staphylococcus aureus* against imipenem, vancomycin, and gentamycin. This finding corresponds to a previous study that also found that *Staphylococcus aureus* was susceptible to higher generation of antibiotics ^19^. The high sensitivity to gentamycin has also been reported by other authors as well ^20^. We found that *Staphylococcus aureus* is usually resistant to various antibiotics and the infection might be acquired in the hospital.

Among the Gram-negative bacteria, *Escherichia coli* was found to be susceptible to ceftriaxone, cefotaxime, gentamycin, cefixime, ceftazidime, and cefuroxime. Furthermore, we found that *Escherichia coli* were less sensitive to cloxacillin with a frequency of 47.8%. Among three isolated *Klebsiella* spp., all organisms were resistant to cephradine, penicillin, cloxacillin, cefuroxime, tetracycline, and ciprofloxacin. Similarly, Okonko *et al*. ^21^ had observed a high level of resistance by *Klebsiella* spp. to most antibiotics. However, they noticed that all three *Klebsiella* spp. Isolates were susceptible to gentamycin and ceftazidime. This high susceptibility pattern might support gentamycin as a suitable antibiotics to treat *Klebsiella* infection ^22^. Among eight isolated *Pseudomonas* spp., all were resistant to cephradine, penicillin, cloxacillin, cefuroxime, amoxicillin, and cefotaxime in this study. We found that five *Pseudomonas* isolates were susceptible to ceftriaxone, four were susceptible to imipenem and gentamycin, and three were susceptible to tetracycline, ciprofloxacin, azithromycin, and ceftazidime. Only one *Pseudomonas* spp. isolate was susceptible to co-trimoxazole, cefixime, and nitrofurantoin.

The susceptibility pattern that we found indicates that most of the isolated strains were multi-drug resistant. Similarly, a study conducted in European setting reported a high resistance of *Pseudomonas* spp., mostly isolated from surgical wounds ^23^. Several previous studies carried out in different settings also support the multi-drug resistance pattern of *Pseudomonas* spp. ^24– 26^. The mechanisms of intrinsic resistance of *Pseudomonas* spp. over most of the antimicrobial agents has emerged because of the low permeability of its outer membrane and the naturally occurring chromosomal Amp β-lactamase ^27, 28^.

The control of wound infections is becoming difficult due to widespread bacterial resistance to antibiotics. Previous studies also notified an increased incidence of bacterial infections by methicillin-resistant *Staphylococcus aureus*, polymicrobial flora and different fungi ^29^. As wound infections are found to be common in this study, prior knowledge of the causative agents of can be a helpful tool in selecting the empiric antimicrobial therapy to control infection

In developing countries, physicians generally do not wait for the culture reports and sometimes, there may be a delay in conducting or reporting of a culture sensitivity test. Hence, with our study, we would like to urge the physicians to start an empirical therapy with a combination of either of the following as an empirical treatment regime:

1) Azithromycin/Imipenem and Ceftriaxone;

2) Gentamycin and Imipenem/Ceftriaxone

3) Ceftazidime and Imipenem.

After application of the above mentioned combination regime, culture sensitivity is advised to be performed in next step. Irrespective of the report, the entire course should be completed and if the condition still remains has not improved, urgent change of treatment plan according to the culture sensitivity report should be carried out.

We would discourage the use of penicillin and amoxicillin, since the resistance towards them has been on the rise. We would also urge physicians to not to prescribe the last resort drugs like vancomycin and linezolid, since they should be used as only in high resistance cases.

### Study limitations

The susceptibility patterns of bacterial isolates to commonly prescribed antibiotics like ceftriaxone, cefuroxime, ciprofloxacin, and azithromycin might not be generalized globally. The fact that our research was a single center study and had a small sample size were other drawbacks. However, our result might represent the scenario of a developing country. Moreover, high-quality data and laboratory support were the particular strengths of this study.

## Conclusions

The most common isolate in wound infection was *Staphylococcus aureus,* followed by *Escherichia coli*, *Pseudomonas* spp., *Klebsiella* spp., and *Streptococcus pyogenes*. Gram-negative bacteria were sensitive to fewer than thirty percent of the commonly prescribed antibiotics, which can be a matter of great concern when treating wound infections. The judicious use of antibiotic prophylaxis and reporting can be the most effective means to reduce the wound infection rate.

## Data availability

The data referenced by this article are under copyright with the following copyright statement: Copyright: © 2017 Roy S et al.

Data associated with the article are available under the terms of the Creative Commons Zero "No rights reserved" data waiver (CC0 1.0 Public domain dedication). 

**Dataset 1: Patient characteristics.** Age, sex, residence, occupation, blood pressure, and diabetic mellitus status are given. DOI, 10.5256/f1000research.12887.d185740 ^30^.

**Dataset 2: Antibiotic susceptibility of bacterial cultures.** Includes data on *S. aureus, S. pyogenes, E. coli, Klebsiella spp, Pseudomonas,* and *Proteus.* DOI, 10.5256/f1000research.12887.d185741 ^31^.

## References

[1] Insan NG Payal N Singh M: Post operative wound infection: Bacteriology and antibiotic sensitivity pattern. *International Journal of Current Research and Review.* 2013;5(13):74–79. Reference Source

[2] Howard R Lee J: Surgical wound infections: epidemiology, surveillance, and clinical management. *Surgical Infectious Diseases.* 1995;401–12.

[3] Bowler PG Duerden BI Armstrong DG: Wound microbiology and associated approaches to wound management. *Clin Microbiol Rev.* 2001;14(2):244–69. 10.1128/CMR.14.2.244-269.2001 11292638 PMC88973

[4] Lilani S Jangale N Chowdhary A: Surgical site infection in clean and clean-contaminated cases. *Indian J Med Microbiol.* 2005;23(4):249–52. 16327121

[5] Haley RW Culver DH White JW: The nationwide nosocomial infection rate. A new need for vital statistics. *Am J Epidemiol.* 1985;121(2):159–67. 10.1093/oxfordjournals.aje.a113988 4014113

[6] Mustafa A: Incidence of nosocomial wound infection in postoperative patients at a teaching hospital in Kashmir. *JK— Practitioner.* 2004;2(1):38–4.

[7] Hussain T Fazal M Ahmed A: Nosocomial infection-A cross-sectional study in the surgical wards of Dhaka Medical College Hospital. *J Prev Soc Med.* 1991;10:70–3.

[8] Koontz FP: Trends in post-operative infections by Gram-positive bacteria. *Int J Antimicrob Agents.* 2000;16 Suppl 1:S35–7. 10.1016/S0924-8579(00)00304-6 11137407

[9] Emmerson AM Enstone JE Griffin M: The Second National Prevalence Survey of infection in hospitals--overview of the results. *J Hosp Infect.* 1996;32(3):175–90. 10.1016/S0195-6701(96)90144-9 8690881

[10] Zaman SB Hussain MA Nye R: A Review on Antibiotic Resistance: Alarm Bells are Ringing. *Cureus.* 2017;9(6):e1403. 10.7759/cureus.1403 28852600 PMC5573035

[11] Cheesbrough M: District laboratory practice in tropical countries. Cambridge university press;2006 Reference Source

[12] CLSI C: Performance Standards for Antimicrobial Susceptibility Testing: Twentieth Informational Supplement. CLSI Document M100-S20, Clinical and Laboratory Standards Institute, Wayne, Pa USA;2010;30.

[13] Zaman SB Hussain MA Hossain N: Antibiotic Resistance: A Tragedy of the Common. *International Journal of Research Studies.* 2017;1(2):7–9. Reference Source

[14] Raza MS Chander A Ranabhat A: Antimicrobial susceptibility patterns of the bacterial isolates in post-operative wound infections in a tertiary care hospital, Kathmandu, Nepal. *Open Journal of Medical Microbiology.* 2013;3(3):159 10.4236/ojmm.2013.33024

[15] Dionigi R Rovera F Dionigi G: Risk factors in surgery. *J Chemother.* 2001;13(Spec No 1(1)):6–11. 10.1179/joc.2001.13.Supplement-2.6 11936382

[16] Nwachukwu NC Orji FA Okike UM: Antibiotic susceptibility patterns of bacterial isolates from surgical wounds in Abia State University Teaching Hospital (ABSUTH), Aba–Nigeria. *Research Journal of Medicine and Medical Sciences.* 2009;4(2):575–9. Reference Source

[17] Afroz H Fakruddin M Masud MR: Incidence of and risk factors for Hospital Acquired Infection in a Tertiary Care Hospital of Dhaka, Bangladesh. *Bangladesh Journal of Medical Science.* 2017;16(3):358–69. 10.3329/bjms.v16i3.32847

[18] Aman S: Bacteriological analysis of wound infection in Mayo hospital, Lahore. *J Pak Med Assoc.* 1982;32(3):66–68. 6808183

[19] Mengesha RE Kasa BG Saravanan M: Aerobic bacteria in post surgical wound infections and pattern of their antimicrobial susceptibility in Ayder Teaching and Referral Hospital, Mekelle, Ethiopia. *BMC Res Notes.* 2014;7(1):575. 10.1186/1756-0500-7-575 25164127 PMC4158133

[20] Zaman SB Hossain N Yasir Arafat SM: Management of Newborn Infection: Knowledge and attitude among health care providers of selected sub-district hospitals in Bangladesh. *International Journal of Perceptions in Public Health.* 2017;1(2):127–32. Reference Source

[21] Okonko IO Soleye FA Amusan TA: Incidence of multi-drug resistance (MDR) organisms in Abeokuta, Southwestern Nigeria. *Global Journal of Pharmacology.* 2009;3(2):69–80. Reference Source

[22] Abe-Aibinu IE Ohaegbulam V Odugbemi TO: A comparative study on the antimicrobial susceptibility patterns of *Klebsiella* and Enterobacter species from the Lagos university teaching hospital. *Journal of the Nigerian Infection Control Association.* 2000;3(2):14–7. 10.4314/jnica.v3i2.10720

[23] Fluit AC Jones ME Schmitz FJ: Antimicrobial susceptibility and frequency of occurrence of clinical blood isolates in Europe from the SENTRY antimicrobial surveillance proGram, 1997 and 1998. *Clin Infect Dis.* 2000;30(3):454–60. 10.1086/313710 10722427

[24] Daini OA Effiong MJ Ogbolu OD: Quinolones Resistance and R-Plasmids of clinical isolates of *Pseudomonas* species. *Sudan JM Sci.* 2008;3(2):139–46. 10.4314/sjms.v3i2.38528

[25] Sekiguchi J Asagi T Miyoshi-Akiyama T: Multidrug-resistant *Pseudomonas aeruginosa* strain that caused an outbreak in a neurosurgery ward and its aac(6')-Iae gene cassette encoding a novel aminoglycoside acetyltransferase. *Antimicrob Agents Chemother.* 2005;49(9):3734–42. 10.1128/AAC.49.9.3734-3742.2005 16127047 PMC1195402

[26] Nordmann P Guibert M: Extended-spectrum beta-lactamases in *Pseudomonas aeruginosa*. *J Antimicrob Chemother.* 1998;42(2):128–31. 9738828 10.1093/jac/42.2.128

[27] Sexton DJ: The impact of antimicrobial resistance on empiric antibiotic selection and antimicrobial use in clinical practice. *J Med Liban.* 2000;48(4):215–20. 11214192

[28] Olayinka AT Olayinka BO Onile BA: Antibiotic susceptibility and plasmid pattern of *Pseudomonas aeruginosa* from the surgical unit of a university teaching hospital in north central Nigeria. *International Journal of Medicine and Medical Sciences.* 2009;1(3):079–83. Reference Source

[29] Shittu AO Kolawole DO Oyedepo EA: A study of wound infections in two health institutions in Ile-Ife, Nigeria. *Afr J Biomed Res.* 2002;5(3):97–102. 10.4314/ajbr.v5i3.53994 12732758

[30] Roy S Ahmed MU Uddin BMM: Dataset 1 in: Evaluation of antibiotic susceptibility in wound infections: A pilot study from Bangladesh. *F1000Research.* 2017 Data Source 10.12688/f1000research.12887.1 PMC5820593 29527295

[31] Roy S Ahmed MU Uddin BMM: Dataset 2 in: Evaluation of antibiotic susceptibility in wound infections: A pilot study from Bangladesh. *F1000Research.* 2017 Data Source 10.12688/f1000research.12887.1 PMC5820593 29527295

