# Supplementary material for: Evaluation of antibiotic susceptibility in wound infections: A pilot study from Bangladesh
Source: F1000Res. 2017 Dec 7;6:2103. [Version 1] doi: 10.12688/f1000research.12887.1 (PMC5820593; doi:10.12688/f1000research.12887.1)
Supplement: Peer review history

# Peer review: Referee response for version 1

**DOI:** 10.5256/f1000research.13969.r30324

**Published:** 2018-02-19

**Authors:** Mohammad Siddiqur Rahman Khan

**Affiliations:** 1 Department of Infectious Diseases and Pathology, Univeristy of Florida, Gainesville, FL, USA

This manuscript is suitable to be indexed after some minor corrections.

 Methods:

 The title should be Materials and Methods.

 Introduction:

 The source of infection was not clear from the description. That will be a valuable information and help others to minimize the hospital-acquired infection. 

 Data collection:

 Specimen transportation temperature was not mentioned.

I have read this submission. I believe that I have an appropriate level of expertise to confirm that it is of an acceptable scientific standard.

# Peer review: Referee response for version 1

**DOI:** 10.5256/f1000research.13969.r29451

**Published:** 2018-01-22

**Authors:** Md. Abu Sayeed

**Affiliations:** 1 Department of Clinical and Molecular Sciences, Polytechnic University of Marche, Ancona, Italy

Authors studied the prevalence of different bacterial pathogens and their antibiotic susceptibility in various types of wound infections. They used 105 samples. They studied colony morphology, and performed Gram-staining and standard biochemical tests to identify the bacteria. Authors used the disc diffusion method for assessing antibiotic susceptibility. Authors found 62.9% Gram-positive and 37.1% Gram-negative bacteria in their samples.They found *Staphylococcus aureus *(55.7%), *Escherichia coli *(23.7%), *Pseudomonas* spp. (8.2%), *Streptococcus pyogenes* (7.2%), Klebsiella spp. (3.1%), and *Proteus* spp. (2.1%). Authors stated that *Staphylococcus* *aureus* (>50%) was sensitive to all antibiotics they used, while *Streptococcus pyogenes* (>50%) was sensitive to all antibiotics except co-trimoxazole. *Escherichia coli* (>50%) was sensitive to co-trimoxazole, cefixime, aztreonam, cefuroxime, tetracycline, imipenem, ceftriaxone, cefotaxime, gentamycin, ceftazidime, nitrofurantoin; *Klebsiella* sp. (>60%) was sensitive to azithromycin, gentamycin, ceftazidime; *Pseudomonas* sp. (>50%) was sensitive to imipenem, ceftriaxone, gentamycin; *Proteus* sp. (>50%) was sensitive to all antibiotics except cephradine, penicillin, cloxacillin, amoxicillin.

 This manuscript is suitable for indexing after minor revision. My suggestions are here:

 -English needs revision.

**Abstract section**

 - *Methods:* The headline of this paragraph should be Materials and Methods.

 - *Results:* This section seems incomprehensive. For example, authors stated the percentage of four isolates only where they found six, authors should also include the other two isolates. Authors only stated the sensitivity of gram-positive bacteria to different antibiotics; authors should also state the sensitivity of gram-negative bacteria.

 - *Conclusions:* based on the study objectives, what is the significance of the study?

**Introduction section**

 -Lines 11-14: should provide reference.

 -Lines 14-15: need more appropriate reference.

 -Lines 18-20: should provide recent information and the reference.

 -Lines 31-32: surgical wound infections rate should be corrected based on the cited reference.

**Methods section**

 -The headline of this paragraph should be Materials and Methods.

 - *Data collection: *Did authors use the ‘commercially prepared paper disc’ of all the antibiotics or prepare in their laboratory? Authors should mention the company name of all antibiotics. Authors should mention which biochemical tests they performed.

**Results section**

 - *Isolation of different types of bacteria: *should mention the information of all isolated bacteria.

 -Isolation of different types of bacteria and Sensitivity pattern of isolated Gram-positive and Gram-negative bacteria: if authors would include some photographs of ‘biochemical tests’ and ‘sensitivity tests’ that would definitely increase the strength of the article, even without photographs the manuscript is ok.

I have read this submission. I believe that I have an appropriate level of expertise to confirm that it is of an acceptable scientific standard.

# Response

**Published:** 2018-02-02

**Authors:** Varshil Mehta

**Affiliations:** MGM Medical College, India

Thank you for your comments and expert opinion. We will certainly update the article as per your suggestions.
